# Secondary Necrosis Following Caspase‐Activation can Occur Independently of Gasdermin E

**DOI:** 10.1002/advs.202507381

**Published:** 2025-11-04

**Authors:** Shubhangi Gavali, Francesca Maremonti, Wulf Tonnus, Florian Gembardt, Marlena Nastassja Schlecht, Karolin Flade, Anne von Mässenhausen, Stephen W. G. Tait, Stefan R. Bornstein, Andreas Linkermann

**Affiliations:** ^1^ Department of Medicine V University Medical Centre Mannheim University of Heidelberg 68167 Mannheim Germany; ^2^ Department of Internal Medicine III University Hospital Carl Gustav Carus at the Technische Universität Dresden 01307 Dresden Germany; ^3^ Institute of Clinical Chemistry and Clinical Pharmacology University Hospital Bonn 53127 Bonn Germany; ^4^ Cancer Research UK Scotland Institute Glasgow 461 1BD UK; ^5^ Diabetes and Nutritional Sciences King's College London London WC2R 2LS UK; ^6^ Center for Regenerative Therapies Technische Universität Dresden 01307 Dresden Germany; ^7^ Paul Langerhans Institute Dresden of Helmholtz Centre Munich at University Clinic Carl Gustav Carus of TU Dresden Faculty of Medicine 01307 Dresden Germany; ^8^ Division of Nephrology Department of Medicine Albert Einstein College of Medicine Bronx NY 10467 USA

**Keywords:** B cell lymphoma 2 (BCL2), death receptor, immunotoxins, regulated necrosis

## Abstract

Regulated necrosis is a known direct consequence of activation of the necroptosis‐ and pyroptosis‐ pathways, but may also result from apoptosis in a process referred to as secondary necrosis. Apoptosis is well understood to be mediated by caspase activation, but the mechanisms that lead to plasma membrane rupture in secondary necrosis remain considerably obscure. Recent data suggested a caspase‐mediated cleavage of gasdermin E (GSDME), a member of the gasdermin family. Here, apoptosis induced by diphtheria toxin (DT) is employed as a novel tool to study secondary necrosis. In addition, cisplatin and anti‐Fas monoclonal antibody Jo2 are employed to study secondary necrosis in cell culture and in vivo, respectively. Despite prominent, yet epiphenomenal cleavage of GSDME, it is demonstrated that silencing or CRISPR/Cas9‐mediated knockout of GSDME does not compromise the kinetics of secondary necrosis induced by DT or cisplatin. During Jo2‐induced acute liver toxicity in mice, GSDME expressed in the necrotic liver is detected predominantly in its uncleaved form. In conclusion, the hypothesis of GSDME to be a central mediator of secondary necrosis in these model systems is disproved.

## Graphical abstract

Secondary necrosis follows apoptosis, but its mechanism is unclear. This study shows that although gasdermin E is cleaved during this process, it is not required for membrane rupture. Across multiple models, secondary necrosis occurred regardless of gasdermin E presence, challenging prior claims and redefining the understanding of how apoptotic cells progress to necrosis.

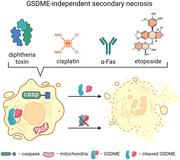

## 1 Introduction

Apoptosis is a well‐characterized cell death pathway that is mediated by caspases and encompasses typical features such as phosphatidylserine exposure on the outer leaflet of the plasma membrane and its blebbing.^[^ ^1^ ^]^ Intrinsic apoptosis involves mitochondrial outer membrane permeabilization (MOMP), formation of the apoptosome comprised of caspase‐9, cytochrome c, and apoptotic protease activating factor‐1 (Apaf‐1) and the caspase‐9‐mediated cleavage and activation of caspase‐3. MOMP is controlled by members of the B cell lymphoma 2 (BCL2) family such as BCL2 associated X protein (BAX), BCL2 homologous antagonist/killer (BAK), and myeloid cell leukemia 1 (MCL1). In contrast to signaling pathways of regulated necrosis, such as necroptosis or pyroptosis, plasma membrane rupture as a secondary event following caspase activation does not occur immediately, but involves an ill‐defined process referred to as “secondary necrosis” after apoptosis.^[^ ^2^, ^3^ ^]^ Two recent studies have indicated a role for the caspase‐3 target gasdermin E (GSDME) as an essential mediator of secondary necrosis.^[^ ^4^, ^5^ ^]^

Diphtheria is a potentially life‐threatening childhood disease that, before vaccination mostly eradicated this condition, presents with swelling of the palate, sore throat, stridor with breathing and swallowing difficulty and a husky voice. Such symptoms clinically present as the result of necrotic lesions in the upper airway epithelia. The mechanism of action of diphtheria toxin (DT) involves the inhibition of eukaryotic protein synthesis including modification of diphthamide, a post‐translationally modified histidine amino acid on the human eukaryotic elongation factor 2 (eEF2). Diphthamide is the target of the ADP‐ribosylating DT that leads to the death of the toxin‐targeted cell. Early reports in the 1990s have suggested DT to mediate apoptosis and ADP ribosylation to be required for DNA fragmentation and cell lysis (secondary necrosis).^[^ ^6^ ^]^ Others reported that the loss of diphthamide sensitizes MCF7 cells to tumor necrosis factor (TNF) receptor‐mediated apoptosis.^[^ ^7^ ^]^ Similar to DT, Pseudomonas exotoxin A also targets eEF2 and has been described to be BAX‐dependent and associated with the degradation of Mcl‐1.^[^ ^8^ ^]^ Mechanisms of secondary necrosis following DT application, however, remain incompletely understood, and while this study focuses on gasdermin E (GSDME), the potential involvement of other gasdermins (e.g., GSDMA, GSDMB, GSDMC, GSDMD, and Pejvakin) remains unexplored.

Here, we demonstrate that DT‐mediated necrosis is a form of secondary necrosis that involves BAX/BAK‐mediated apoptosis and can be effectively prevented using caspase inhibitors. The transition from apoptosis to secondary necrosis, as assessed by plasma membrane rupture, is associated with cleavage of GSDME. However, CRISPR/Cas9‐mediated deletion of GSDME does not influence the necrotic phenotype in any way. We, therefore, assessed GSDME‐cleavage in other models of secondary necrosis, such as Fas (CD95, Apo‐1)‐mediated acute liver failure model^[^ ^9^ ^]^ which was not associated with GSDME cleavage. In summary, DT‐mediated cell death is caspase‐dependent but does not require GSDME cleavage for progression to secondary necrosis. Similarly, Fas‐mediated necrotic liver injury does not involve GSDME cleavage. We therefore conclude that GSDME is not required for secondary necrosis in vitro and in vivo in these systems. Finally, we identify caspase‐inhibition as a strategy to partially prevent DT‐mediated cell death that could potentially be therapeutically harnessed upon infection. However, we acknowledge that these findings are based on HeLa and CD10 cells, and further validation in additional cell models is necessary to fully establish the therapeutic potential of caspase inhibition.

## 2 Results

**Figure 1:**
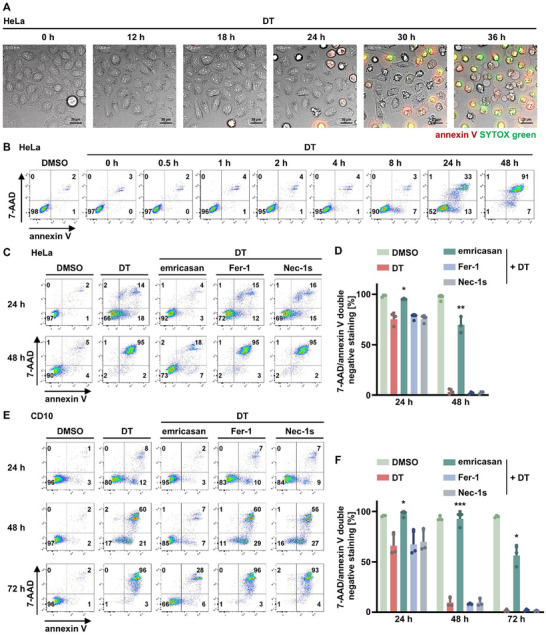
Caspase inhibitors prevent secondary necrosis following DT application. A) Still images of a time‐lapse video (Video S2, Supporting Information) of 10 nm DT‐treated HeLa cells demonstrating the dynamics of cell death by annexin V binding and SYTOX green uptake. B) HeLa cells were treated for 48 h with DT. Representative scatter plots show induction of cell death by 7‐aminoactinomycin (7‐AAD) and annexin V positivity. C) HeLa cells were treated for 48 h with DT in presence of 5 µm emricasan, 1 µm Fer‐1, or 10 µm Nec‐1s. D) Quantification of 7‐AAD/annexin V double‐negative populations of HeLa cells from (C). E) CD10 cells were treated for 72 h like in (C). F) Quantification of 7‐AAD/annexin V double‐negative populations of CD10 cells from (E). Data represent mean ± SD (*n* = 3); *p < 0.05, **p < 0.01, ***p < 0.001.

**Figure 2:**
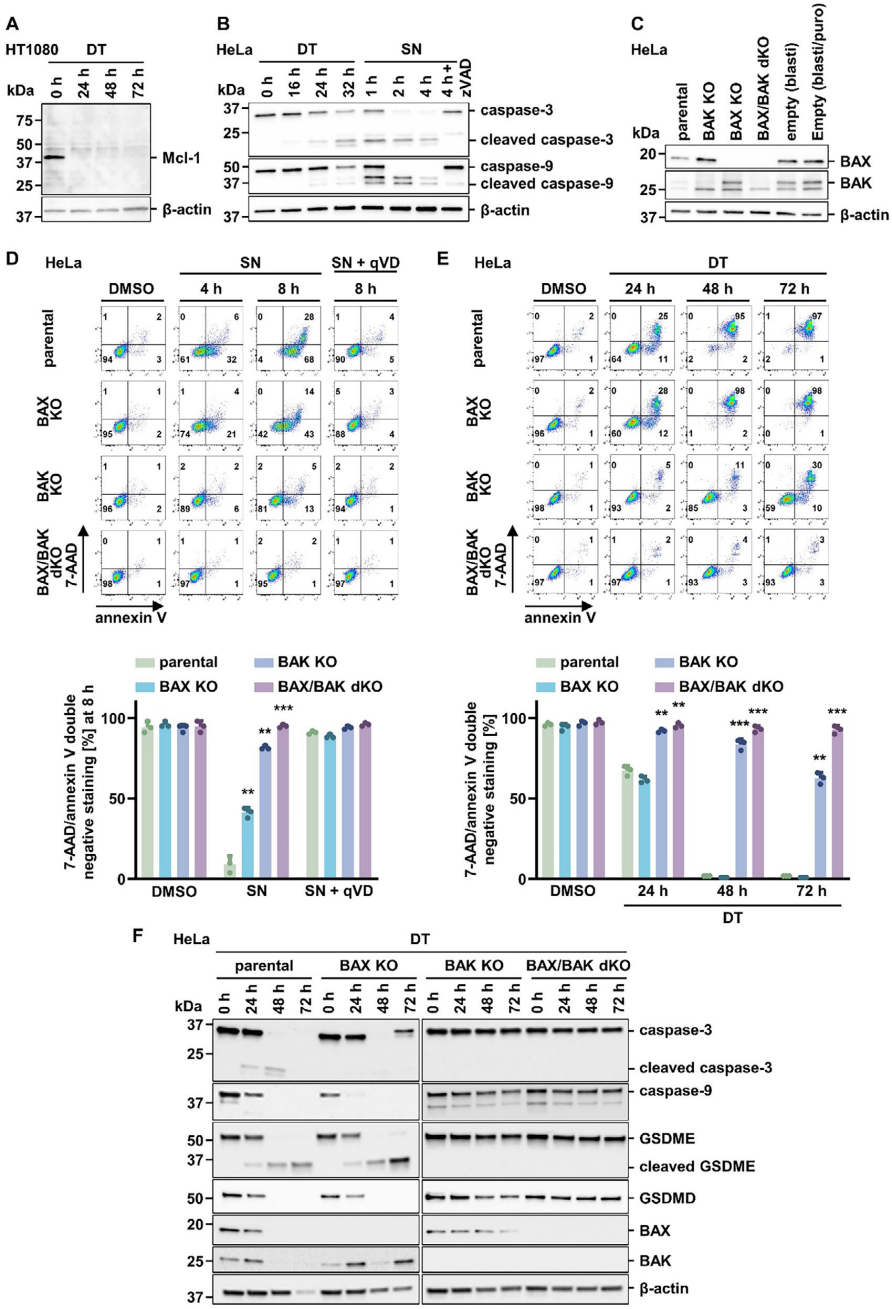
BAX/BAK knockout HeLa cells are resistant to DT‐mediated death. A) HT1080 cells were treated for 72 h with 10 nm DT before investigating protein levels of Mcl‐1 by Western blotting. β‐actin serves as a loading control. B) Protein expression of caspase‐3/‐9 in HeLa cells treated with DT for 32 h or with a combination of 5 µm S63845 (MCL1‐inhibitor) and 5 µm of navitoclax (combined BCL2/BCL‐xL‐inhibitor, referred to as SN treatment) for 4 h. C) Protein expression levels of BAX and BAK for knockout (KO) validation in HeLa cells. D) Upper panel: parental, BAX KO, BAK KO, and BAX/BAK double KO (dKO) HeLa cells treated with SN with or without 20 µm of the caspase inhibitor qVD for 8 h. Lower panel: Quantification of 7‑AAD/annexin V double negative populations from the upper panel. E) Upper panel: parental, BAX KO, BAK KO, and BAX/BAK dKO HeLa cells were treated with DT for 72 h. BAX/BAK dKO prevented HeLa cells from DT‐mediated cell death and cell death was assessed by flow cytometry. Lower panel: Quantification of 7‑AAD/annexin V double‐negative populations from the upper panel. F) Western blot showing protein expression levels of caspase‐3/‐9, GSDME, GSDMD, BAX, and BAK in indicated cells treated with DT for 72 h. Data represent mean ± SD (*n* = 3); **p < 0.01, ***p < 0.001.

To assess the morphological features of diphtheria toxin (DT)‐induced cell death, we started by recording a time‐lapse video of unstimulated (Video S1, Supporting Information) and DT‐treated HeLa cells alongside with a staining for annexin V and SYTOX green (**Figure** **1**A and Video S2, Supporting Information). As expected, we observed a classical apoptotic sequence characterized by plasma membrane blebbing following a phase of cellular shrinkage, similar to default intrinsic apoptosis which includes mitochondrial outer membrane permeabilization (MOMP), induced by combined treatment with the MCL1‐inhibitor S63845 and the combined BCL2/BCL‐xL inhibitor navitoclax (referred to as SN‐treatment (Video S3, Supporting Information)). In parallel, we employed flow cytometry analyses to investigate cellular positivity for annexin V and 7‐aminoactinomycin (7‐AAD) following DT treatment for 48 h. While annexin V positivity was observed as early as 8 h following DT treatment, positivity for 7‐AAD (necrosis) occurred after 24 h (34%) and was almost complete after 48 h (92%) (Figure 1B). We confirmed that this type of necrosis is not mediated by ferroptosis or necroptosis by addition of the ferroptosis inhibitor ferrostatin‐1 (Fer‐1) and the receptor‐interacting serine/threonine protein kinase 1 (RIPK1) inhibitor necrostatin‐1s (Nec‐1s), respectively (Figure 1C,D). Besides HeLa cells, we observed a similar pattern of DT‐induced secondary necrosis in the human kidney tubular cell line CD10 (Figure 1E,F). These experiments formally ruled out a contribution of ferroptosis and necroptosis during this process. However, we additionally investigated the default ferroptosis cell line HT1080 upon DT treatment and observed depletion of the BCL2 family member MCL1 (**Figure** **2**A), prompting us to investigate the intrinsic branch of the apoptotic cascade. Indeed, we observed DT‐induced cleavage of caspase‐9 and caspase‐3 in a HeLa cell assay controlled by SN‐treatment (Figure 2B). The pan‐caspase inhibitor zVAD‐fmk (zVAD) functions as a protection control. These data indicated an involvement of the pro‐apoptotic BH3‐only proteins BAX and BAK. We therefore generated CRISPR/Cas9 knockout cell lines (BAX KO, BAK KO, and BAX/BAK dKO) from parental HeLa cells (Figure 2C). We confirmed the appropriate function of these cells by SN‐treatment over time, with pan‐caspase inhibitor qVD‐OPH (qVD) as protection control (Figure 2D). As demonstrated in Figure 2E, only the absence of both BAX and BAK reversed the cell death phenotype induced by DT, while BAX KO cell line exhibited >70% annexin V/7‐AAD double positivity after 72 h, and BAK KO cell line exhibited partial protection against DT‐induced cell death. Collectively, these experiments confirmed critical involvement of the intrinsic apoptotic pathway before the onset of secondary necrosis.

**Figure 3:**
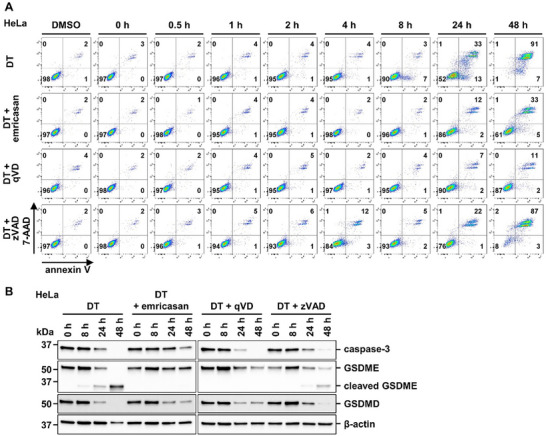
GSDME cleavage kinetics in DT‐treated HeLa cells. A) HeLa cells were treated for 48 h with 10 nM DT in the presence of 5 µM emricasan, 20 µM qVD, or 20 µM zVAD. Representative scatter plots show 7‐AAD‐ and annexin V‐positivity. B) Protein expression levels of caspase‐3, GSDME, and GSDMD in HeLa cells treated as in (A). β‐actin serves as a loading control.

It was recently demonstrated that secondary necrosis can be mediated by caspase‐3‐dependent cleavage of the pore‐forming gasdermin family member gasdermin E (GSDME). We, therefore, investigated potential GSDME cleavage in our experimental setup. Interestingly, DT caused complete GSDME cleavage within 48 h in parental HeLa cells. Surprisingly, the GSDME cleavage was absent in combined BAX‐ and BAK‐deficient cells (Figure 2F). We, therefore, decided to investigate a time course of both DT‐induced cellular necrosis and GSDME cleavage in the presence of the pan‐caspase inhibitors emricasan, qVD and zVAD. We observed that zVAD failed significantly to protect from double positivity for annexin V and 7‐AAD 48 h following DT stimulation, while emricasan and, most prominently, qVD prevented the cells from dying (**Figure** **3**A). Accordingly, GSDME cleavage was observed in the controls and the zVAD‐treated cells and not upon co‐incubation with emricasan or qVD, as assessed by western blotting (Figure 3B). Additionally, we did not observe other obvious changes in the protein content of these cells as visually assessed from Ponceau stainings (Figure S1, Supporting Information). These experiments confirmed the previously reported cleavage of GSMDE downstream of caspase‐3 activation.

**Figure 4:**
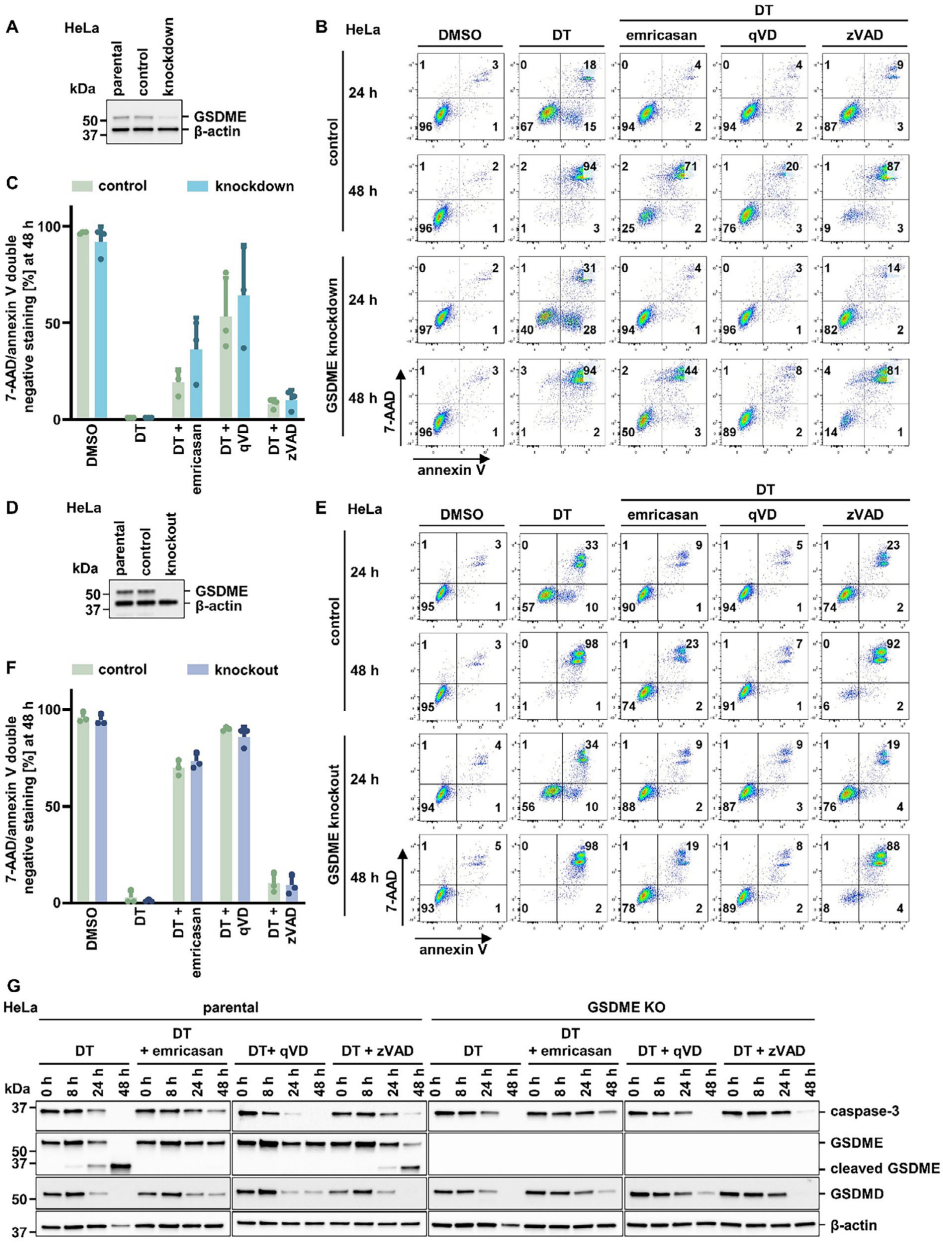
GSDME silencing or genetic knockout does not influence DT‐induced secondary necrosis. A) Western blot analysis to determine knockdown efficacy of GSDME 72 h after transfecting parental HeLa cells with non‐targeting siRNA (control) and GSDME siRNA (knockdown). β‐actin serves as a loading control; GSDME antibody probing was performed without stripping the β‑actin from the blot. B) Control and GSDME knockdown cells were treated with 10 nm DT in the presence of 5 µm emricasan, 20 µm qVD or 20 µm zVAD and assessed by flow cytometry. C) Quantification of 7‑AAD/annexin V double‐negative populations of HeLa cells from (B). D) Western blot analysis to control for GSDME knockout (KO) efficacy as indicated. β‐actin serves as a loading control; GSDME antibody probing was performed without stripping the β‑actin from the blot. E) GSDME KO cells were treated with DT in the presence of caspase‐inhibitors as in (B). F) Quantification of 7‑AAD/annexin V double‐negative populations of HeLa cells from (E). G) Western blot analysis of parental and GSDME KO HeLa cells treated with DT in the presence of caspase inhibitors, showing protein levels of caspase‑3, GSDME, and GSDMD, with β‑actin as a loading control. Data represent mean ± SD (*n* = 3).

To test if GSDME is required for secondary necrosis after DT treatment, we knocked down GSDME and additionally generated CRISPR/Cas9‐knockout cell lines lacking GSDME. The GSDME knockdown efficacy 72 h following co‐incubation with the siRNA is demonstrated in **Figure** **4**A. However, indistinguishable from controls, complete necrosis was observed 48 h after DT stimulation following the GSDME knockdown (Figure 4B,C). Similarly, the GSDME CRISPR/Cas9‐knockout cell line (Figure 4D) failed to prevent DT‐induced secondary necrosis (Figure 4E,F; Figure S2A,B, Supporting Information). Western blotting showed caspase‐3 activation and GSDME cleavage in DT‐treated parental cells, while caspase‐3 activation persisted in GSDME KO cells (Figure 4G). The cleavage pattern of GSDMD was similar in parental and GSDME KO cells, and together with the comparable cell death kinetics, this rules out compensatory involvement of GSDMD in the absence of GSDME. These data suggest that GSDME cleavage represents a correlation during secondary necrosis, but rules out a causative role.

**Figure 5:**
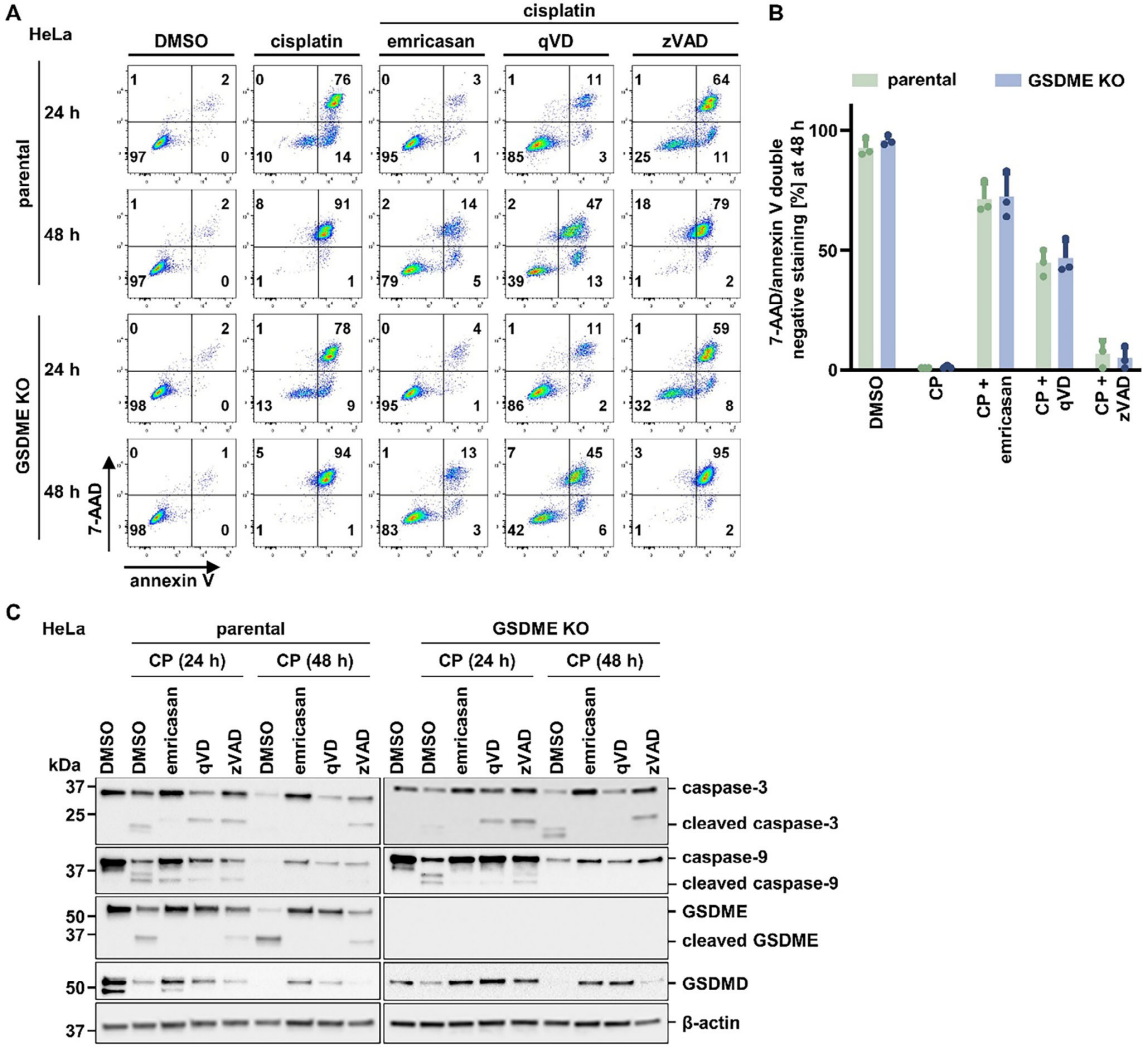
Cisplatin‐induced secondary necrosis does not require GSDME. A) Parental and GSDME KO cells were treated for 48 h with 30 µm cisplatin (CP) in the presence of 5 µm emricasan, 20 µm qVD, or 20 µm zVAD. Representative scatter plots show induction of cell death by 7‐AAD and annexin V positivity. B) Quantification of 7‐AAD/annexin V double‐negative populations from (A). C) Protein expression levels of caspase‐3/‐9, GSDME, and GSDMD in parental and GSDME KO HeLa cells treated as in (A). β‐actin serves as a loading control. Data represent mean ± SD (*n* = 3).

We next established another model of secondary necrosis following caspase activation, stimulated by the DNA‐damaging agent cisplatin. The cisplatin‐induced cell death was associated with caspase‐3 and caspase‐9 cleavage which was prevented by addition of emricasan, and to a lesser extent by qVD and zVAD (**Figure** **5**A–C). Comparable to the DT‐induced secondary necrosis, cisplatin‐induced caspase‐3 and caspase‐9 activation was associated with GSDME cleavage (Figure 5C). However, when we functionally assessed the cisplatin‐induced cell death, secondary necrosis occurred in more than 98% of HeLa cells after 48 h, irrespective of the presence of GSDME (Figure 5A,B). These data indicated that GSDME is not required for secondary necrosis.

To further validate our findings, we examined secondary necrosis induced by etoposide. Treatment for 48 h triggered caspase activation and GSDME cleavage in parental cells, with no difference in cell death kinetics between parental and GSDME KO cells (Figure S3A–C, Supporting Information). These results confirm that GSDME cleavage is not required for etoposide‐induced secondary necrosis.

**Figure 6:**
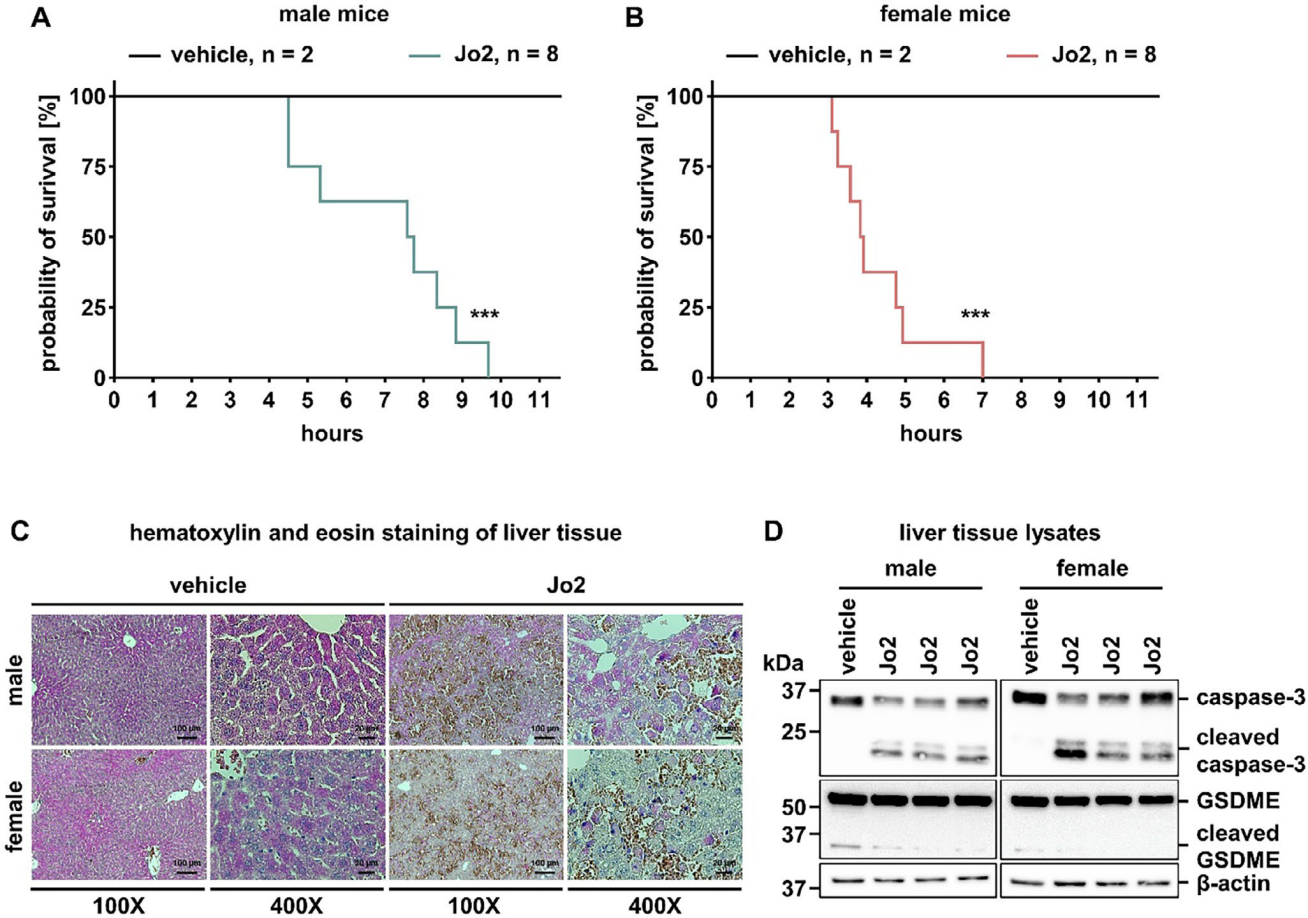
Jo2‐induced liver necrosis in mice is GSDME‐independent. A) Survival plot for male mice (age 8–12 weeks) with intraperitoneal (i.p.) administration of monoclonal agonistic α‐Fas antibody Jo2 (1 mg kg^−1^ body weight) or PBS as vehicle. B) Survival plot for female mice (age 8–12 weeks) with i.p. administration as above. C) Representative hematoxylin and eosin stained liver tissue sections from mice treated as in (A,B). D) Protein expression levels of caspase‐3 and GSDME in liver tissue lysates collected from mice treated as in (A,B). β‐actin serves as a loading control. Statistical analysis was performed using Kaplan–Meier survival analysis. ***p < 0.001.

The anti‐Fas antibody Jo2 causes caspase‐dependent liver necrosis in mice within hours following intraperitoneal injection.^[^ ^9^ ^]^ Because this model represents the most commonly studied approach to the secondary necrosis in vivo, we set out to assess GSDME cleavage in this setting. All male and female C57Bl/6 mice succumbed, as expected, within 10 or 7 h, respectively (**Figure** **6**A,B). In a separate experiment, we collected liver tissues for both histological analysis and lysate preparation at the 3 h time point following Jo2 application. Hematoxylin and eosin staining revealed extensive necrosis, while western blot showed prominent caspase‐3 cleavage in all Jo2‐treated mice (Figure 6C,D). While a residual baseline cleavage of GSDME was detected in both sexes, in the case of Jo2 application, no significant GSDME cleavage was observed (Figure 6D). These data strongly support the above‐mentioned notion that GSDME is not required for secondary necrosis.

## 3 Discussion

It was previously proposed that diphtheria toxin (DT) induces apoptosis based on trypan blue counts of DT‐treated cells, and on inhibition of trypan blue positivity in the presence of pan‐caspase inhibitors.^[^ ^6^, ^10^ ^]^ Today, these methods are obsolete given their poor sensitivity and specificity.^[^ ^1^, ^11^ ^]^ Proper assessment of both apoptosis and secondary necrosis over time by flow cytometry and combined annexin V / 7‐AAD stainings have not been investigated to the best of our knowledge. Therefore, DT‐mediated secondary necrosis has hardly been studied in cell culture. We demonstrate that DT‐mediated secondary necrosis serves as an excellent model to study secondary necrosis following caspase activation. Our data suggest that the clinical presentation in paediatric patients with diphtheria is the pathophysiological result of secondary necrosis. The anti‐Fas (Jo2)‐mediated liver necrosis model^[^ ^9^ ^]^ may serve as the best studied and most severe mouse model of secondary necrosis.^[^ ^12^ ^]^ In contrast to the DT‐induced model, however, this depends on extrinsic receptor‐mediated apoptosis rather than intrinsic apoptosis requiring mitochondrial outer membrane permeabilization (MOMP). In both cases, however, gasdermin E (GSDME) is not required for the cell death/necrosis phenotype. This is in contrast to two recent publications that claimed an indispensable role of GSDME in necrosis after caspase activation. Despite clear cleavage of GSDME following DT‐induced cell death, the GSDME cleavage did not function to cause plasma membrane rupture, as demonstrated in the CRISPR/Cas9 knockout model.^[^ ^4^, ^5^ ^]^ In the Jo2 model, substantial GSDME cleavage was not detected; however, confirming its dispensability using GSDME KO mice remains a limitation of the present study and should be addressed in future work. Collectively, our data indicate that GSDME is dispensable for DT‐induced secondary necrosis. While a subtle modulatory role cannot be excluded, its contribution appears minimal and may have been overstated in prior reports. Cleavage may simply reflect a byproduct of caspase activation due to the presence of an accessible recognition site, rather than a functionally relevant event. Further studies are needed to clarify its mechanistic significance. The differences in this outcome may be explained by different cell lines tested and varying models of apoptosis induction.

Very recently, it has been demonstrated that DT can activate ribotoxic stress and NLR family pyrin domain containing 1 (NLRP1)‐induced pyroptosis.^[^ ^13^ ^]^ Indeed, pyroptosis was reported to occur following treatment with chemotherapeutic drugs in a caspase‐3‐dependent and GSDME cleavage‐mediated pathway to plasma membrane rupture.^[^ ^14^ ^]^ It is interesting to realize that in different cell types, caspase‐mediated GSDME cleavage may or may not be required for plasma membrane rupture. The explanation to such phenomena may be found in future studies by investigating the details of the GSDME pore‐forming machinery.

Promotor‐specific overexpression of the DT receptor (DTR) has been experimentally used in biomedical research to deplete specific cell types in order to analyse and understand their function. Even a tissue‐nonspecific knock‐in of the DTR has been described as the terminator mouse.^[^ ^15^ ^]^ Such models, however, commonly focus on the depletion of the respective cell types as an outcome rather than also considering the influence of potential immunogenicity of the necrotic debris that inevitably occurs during secondary necrosis. This is of particular interest in cases wherein the authors conclude on the suppression of autoimmune phenotypes, such as reported for regulatory T cells^[^ ^16^, ^17^ ^]^ or γδ T cells.^[^ ^18^ ^]^

In conclusion, we demonstrate that DT‐induced cell death may be useful as a model to study secondary necrosis downstream of MOMP. We demonstrate that GSDME cleavage is not required for secondary necrosis downstream of caspase activation and suggest that caspase inhibition may hold promise as a therapeutic option for the treatment of diphtheria.

## 4 Experimental Section

### Reagents

The reagents or resources and compounds and chemicals used in this study are provided in **Table** **1**.

**Table 1:** Reagents or resources and compounds and chemicals.

REAGENT or RESOURCE	SOURCE	IDENTIFIER
Antibodies		
Anti‐GSDME antibody	Abcam	ab215191, ab222407
Anti‐caspase‐3 antibody	Cell Signaling	9662
Anti‐caspase‐9 antibody	Abcam	ab202068
Anti‐MCL1 antibody	Abcam	ab32087
Anti‐BAX antibody	Cell Signaling	2772
Anti‐BAK antibody	Cell Signaling	12105
Anti‐GSDMD antibody	Cell Signaling	97558
Anti‐β‐actin antibody	Cell Signaling	3700S
Anti‐mouse IgG; HRP‐linked antibody	Cell Signaling	7076S
Anti‐rabbit IgG; HRP‐linked antibody	Cell Signaling	7074S
Agonistic α‐Fas antibody Jo2	BD Biosciences	554255

Compounds & chemicals		
DMEM, high glucose, pyruvate	Thermo Fisher Scientific	41966029
Opti‐MEM I Medium	Thermo Fisher Scientific	31985062
Fetal Bovine Serum (FBS)	Thermo Fisher Scientific	10270106
Penicillin‐Streptomycin	Thermo Fisher Scientific	15140122
Blasticidin	InvivoGen	ant‐bl‐1
Puromycin	InvivoGen	ant‐pr‐1
Diphtheria toxin	Merck	D0564‐1MG
S63845	Selleck Chemicals	S8383
Navitoclax (ABT‐263)	Selleck Chemicals	S1001
Cisplatin	Pharmacy of the University Hospital in Dresden	n/a
Etoposide	Merck	341205
Ferrostatin‐1 (Fer‐1)	Merck	341494
7‐Cl‐O‐Nec‐1 (Nec‐1s)	Merck Millipore	5.04297.0001
Emricasan	Sigma Aldrich	SML2227
Q‐VD‐Oph	Selleck Chemicals	S7311
zVAD‐fmk	BD Biosciences	550377
7‐AAD	BD Biosciences	559925
Hematoxylin solution	Merck	H3136
Eosin Y solution	Merck	HT110232
Mounting medium	VectorLabs	H‐5600‐60
Annexin‐V‐FITC	BD Biosciences	556420
Annexin‐V binding buffer	BD Biosciences	556454
SYTOX™ Green	Thermo Fisher Scientific	S7020
Annexin V Alexa Fluor 647 conjugate	Thermo Fisher Scientific	A23204
BCA assay kit	Thermo Fisher Scientific	23225
Ponceau S	Sigma Aldrich	P7170
ECL Prime Western Blotting System	Thermo Fisher Scientific	GERPN2232
Lipofectamine RNAiMAX	Thermo Fisher Scientific	13778075
Silencer Select Negative Control No. 1 siRNA	Thermo Fisher Scientific	4390843
GSDME siRNA	Thermo Fisher Scientific	s230618
TrueCut Cas9 Protein v2	Thermo Fisher Scientific	A36498
Lipofectamine CRISPRMAX Cas9 Transfection Reagent	Thermo Fisher Scientific	CMAX00001
TrueGuide Synthetic sgRNA, DFNA5	Thermo Fisher Scientific	A35533
TrueGuide Synthetic sgRNA, Negative control	Thermo Fisher Scientific	A35526

### Cell Lines and Cell Culture

Human HeLa cells and immortalized human kidney tubular epithelial CD10‐135 cells were kindly provided by Dirk Lindemann and the Rafael Kramann laboratory, respectively. Human HT1080 (Cat# CCL‐121) cells were purchased from the American Type Culture Collection. HT1080 and HeLa cells were cultured in Dulbecco's modified Eagle's medium (DMEM; Thermo Fisher Scientific) supplemented with 10% (v/v) fetal bovine serum (FBS; Thermo Fisher Scientific), 100 U mL^−1^ penicillin, and 100 µg mL^−1^ streptomycin (Pen/Strep, Thermo Fisher Scientific). The CD10‐135 cells were cultured in DMEM:F12 Glutamax (Thermo Fisher Scientific) supplemented with 10% FBS and Pen/Strep. All cell lines were maintained in a humidified 5% CO_2_ atmosphere.

### Cell Death Assay

Cells were seeded in six‐well plates and stimulated with various cell death inducers and inhibitors. Unless otherwise indicated, we used 10 nm diphtheria toxin (DT), 5 µm S63845, 5 µm navitoclax, 30 µm cisplatin, 50 µm etoposide, 1 µm ferrostatin‐1 (Fer‐1), 10 µm necrostatin‐1s (Nec‐1s), 5 µm emricasan, 20 µm Q‐VD‐Oph (qVD), and 20 µm zVAD‐fmk (zVAD). After the indicated time points, the cells were collected and prepared for flow cytometry, western blotting, and imaging.

### Time‐Lapse Imaging

Time‐lapse imaging was performed using a 2.5×/0.12 Fluar objective for the custom‐made 3D‐printed well plated with HeLa cells on an Axiovert 200 M equipped with a large incubation chamber (37°C), 5% CO_2_, and humidity control. Transmitted light and fluorescent images (LED 475‐nm Spectra X light source, emission filter BP 525–550 and LED 631‐nm Spectra X light source, emission filter BP 663–738) were acquired using an Orca flash 4.0 camera. HeLa cells were plated in the above‐described double chamber in DMEM without phenol red (Thermo Fisher Scientific) and treated with vehicle, DT or combination of 5 µm S63845 and 5 µm navitoclax (referred to as SN treatment). Next, 0.5 µM SYTOX Green (Life Technologies), 5 µg mL^−1^ annexin V, Alexa Fluor 647 conjugate (Thermo Fisher Scientific) was added to the cells. The live‐imaging procedure was supported by the Light Microscopy Facility, a core facility of the CMCB Technology Platform at Technische Universität (TU) Dresden.

### Flow Cytometry

Cells were harvested, and the pellets were washed twice in phosphate‐buffered saline (PBS) and stained with 5 µL of 7‐AAD (BD Biosciences) and 5 µL of annexin V–FITC (BD Biosciences) diluted in 100 µL of annexin V–binding buffer (BD Biosciences). After 15 min of incubation, cells were recorded on the LSR II with the FACS Diva 6.1.1 software (BD Biosciences) and subsequently analyzed with the FlowJo v10 software (Tree Star). The flow cytometry procedure was supported by the Flow Cytometry Core Facility of the Center for Molecular and Cellular Bioengineering (CMCB) Technology Platform at TU Dresden.

### Western Blotting

Cells were lysed in ice‐cold 50 mm tris‐HCl (pH 7.5), 150 mm NaCl, 1% NP‐40, 5 mm EDTA supplemented with PhosSTOP (Merck), cOmplete (Merck), and 1 mm phenylmethylsulfonyl fluoride (PMSF) for 30 min on ice. Insoluble material was removed by centrifugation (14 000 g, 30 min, 4 °C). Protein concentration was determined using a commercial bicinchoninic acid (BCA) assay kit according to the manufacturer's instructions (Thermo Fisher Scientific). Equal amounts of protein (typically 25 µg per lane) were resolved on a 4–15% gradient SDS–polyacrylamide gel electrophoresis gel and transferred to a polyvinylidene difluoride membrane (Bio‐Rad). The protein profile was visualized by briefly incubating the membrane in Ponceau S staining Solution. After blocking for 1 h at room temperature, primary antibody incubation was performed overnight at 4°C. Secondary antibodies (anti‐mouse, HRP‐linked antibody, and anti‐rabbit, HRP‐linked antibody, Cell Signaling Technology) were applied at dilution of 1:5000. Proteins were then visualized by enhanced chemiluminescence (Amersham Biosciences).

### siRNA‐Mediated Knockdown of GSDME

HeLa cells were plated in a 10 cm petri dish in 15 mL of antibiotic‐free medium. After 24 h, 180 pmol DFNA5 siRNA and 24 µL of Lipofectamine (Thermo Fisher Scientific) were each mixed in 1.5 mL of Opti‐MEM I Medium (Thermo Fisher Scientific) without serum, combined, and incubated at room temperature for 20 min before dropping the mixture on the cells. The following day, cells were harvested, plated into six‐well plates, and cell death assays were performed as described above. Knockdown efficacy was determined at protein level.

### CRISPR/Cas9‐Mediated GSDME Knockout

HeLa cells were seeded in a six‐well plate in 2 mL Opti‐MEM I Medium. After 24 h, 100 pmol of DFNA5 gRNA or non‐targeting NTgRNA, as control, and Cas9 nuclease solution was prepared with Cas9 Plus reagent. This solution was mixed with pre‐diluted CRISPRMAX reagent in Opti‐MEM I Medium. This Cas9 nuclease/gRNA/transfection reagent complex was incubated at room temperature for 10 min before adding dropwise on the cells. Knockout efficiency was checked at protein level post 7 days of transfection.

### CRISPR/Cas9‐Mediated BAX/BAK Knockout

The LentiCRISPRv2 blast vectors containing guides targeting the human BAX or human BAK gene were kindly provided by Stephen Tait. HEK293T cells were transfected with LentiCRISPRv2 blast vectors and packing plasmids (pspax2, pMD2.G) using ViaFect transfection reagent and medium was changed the next day. 48 h after transfection HT1080 cells were transduced with viral supernatant and then selected using 15 µg mL^−1^ blasticidin and 2 µg mL^−1^ puromycin for BAX and BAK knockout cells respectively. Knockout efficacy was determined at protein level in polyclonal cells.

### Mice

All wild‐type mice (C57Bl/6N) were initially provided by Charles River, Sulzfeld, Germany, at the age of 6–7 weeks. The mice used in the study were kept under stable 12 h circles of darkness and light in the respective facilities. Room temperature was kept between 20 and 24°C and air humidity between 45% and 65%, as documented in daily controls. If not otherwise indicated, all cages were individually ventilated cages in our facility. Approval was granted by the local authorities. Mice had access to sterilized standard pellet food and water *ad libidum*. All cages and nestlets were sterilized by autoclaving before use.

### Jo2‐Induced Liver Failure Mouse Model (TNFRSF6‐Induced Shock)

Monoclonal agonistic α‐Fas (anti‐TNFRSF6, a member of the TNFR superfamily) antibody Jo2 (1 mg kg^−1^ body weight) was administered intraperitonially to mice (age 8–12 weeks). For survival studies, eight mice per group received α‐Fas monoclonal antibody Jo2 and two mice per group received PBS as a vehicle.

In a separate experiment, mice were sacrificed 3 h after injection of Jo2 antibody or vehicle for liver histological analysis and lysate preparation for western blot analysis. The German Approval of Animal Act committee, application No V244‐7224.121‐4 approved the experiments. All in vivo experiments were performed according to institutional, national, and European ethical animal regulations (Protection of Animals Act).

### Hematoxylin and Eosin (H&E) Staining

Fresh liver biopsy tissues were fixed in 4% paraformaldehyde for at least 24 h, then embedded in paraffin. Paraffin sections (4 µm) were deparaffinized in xylene (2 × 5 min) and rehydrated through a graded ethanol series (100%, 95%, 70%, 5 min each). After rinsing in distilled water, sections were stained with hematoxylin (Merck H3136) for 1 min, followed by thorough washing in tap water. Sections were then stained with eosin Y (Merck HT110232) for 2 min, dehydrated through graded ethanol, cleared in xylene, and mounted with VectaMount (H‐5600‐60). Stained sections were analyzed using an Axio Imager or Zeiss Observer Z.1 microscope at 100× and 400× magnifications.

### Statistical Analysis

Statistical analyses were performed using Prism 10 (GraphPad Software, San Diego, CA, USA). Two‐tailed parametric *t*‐tests with Welch's correction were used for single‐group comparisons. Kaplan–Meier analysis was applied for survival curves. Data were considered significant when *p ≤ 0.05, **p ≤ 0.01 or ***p ≤ 0.001.

## Conflict of Interest

The authors declare no conflict of interest.

## Author Contributions

S.G., F.M., W.T., F.G., M.N.S., K.F., N.J.B., and A.M. performed the experiments. S.W.G.T., S.R.B., and A.L. provided essential materials. S.G. and A.L. designed the study and analyzed all primary data. S.G. and A.L. wrote the manuscript.

## Supporting information



Supporting Information

Supplemental Video 3

Supplemental Video 3

Supplemental Video 3

Supplemental Video 1

Supplemental Video 2

Supplemental Video 3

## Data Availability

The data that support the findings of this study are available from the corresponding author upon reasonable request.
