# Supplementary material for: Secondary Necrosis Following Caspase‐Activation can Occur Independently of Gasdermin E
Source: Adv Sci (Weinh). 2025 Nov 4;12(46):e07381. doi: 10.1002/advs.202507381 (PMC12697764; doi:10.1002/advs.202507381)
Supplement: Supplementary file 1 — Supporting Information [file ADVS-12-e07381-s002.docx]

Supplementary Material for

**Secondary Necrosis Following Caspase‐Activation can Occur Independently of Gasdermin E**

**Supplementary Figure S1. Ponceau S staining of HeLa cell lysates demonstrate changes in overall protein content during secondary necrosis.** HeLa cells were treated for 48 h with 10 nM DT with or without 5 µM emricasan, 20 µM qVD or 20 µM zVAD. Protein profile on the transferred membrane visualized using Ponceau S staining.

**Supplementary Figure S2. Cell death kinetics of DT-treated control and GSDME KO HeLa cells. A** Control HeLa cells transfected with non-targeting guide RNA were treated for 48 h with 10 nM DT in the presence of 5 µM emricasan, 20 µM qVD or 20 µM zVAD. Representative scatter plots show positivity for 7-aminoactinomycin (7-AAD) and annexin V. **B** GSDME KO HeLa cells were treated as in (**A**).

**Supplementary Figure S3. Etoposide-induced secondary necrosis does not require GSDME. A.** Parental, GSDME KO, and BAX/BAK-dKO cells were treated for 48 h with 50 µM etoposide. Representative scatter plots show induction of cell death by 7-aminoactinomycin (7-AAD) and annexin V positivity. **B**. Quantification of 7-AAD/annexin V double negative populations from (A). **C**. Protein expression levels of caspase-3/-9, GSDME, and GSDMD in parental, GSDME KO, and BAX/BAK dKO HeLa cells treated as in (A). β-actin serves as a loading control. Data represent mean ± SD (n = 3), ***p < 0.01*.

**Supplementary video S1.** Control time-lapse video of HeLa cells treated with DMSO and stained with annexin V and SYTOX green.

**Supplementary video S2.** Time-lapse video of HeLa cells treated with 10 nM DT and stained with annexin V and SYTOX green.

**Supplementary video S3.** Time-lapse video of HeLa cells treated with 5 µM S63845 (MCL1-inhibitor) and 5 µM of navitoclax (combined BCL2/BCL-xL-inhibitor) and stained with annexin V and SYTOX green.
