# Supplementary figures and images for: Secondary Necrosis Following Caspase‐Activation can Occur Independently of Gasdermin E
Source: Adv Sci (Weinh). 2025 Nov 4;12(46):e07381. doi: 10.1002/advs.202507381 (PMC12697764; doi:10.1002/advs.202507381)

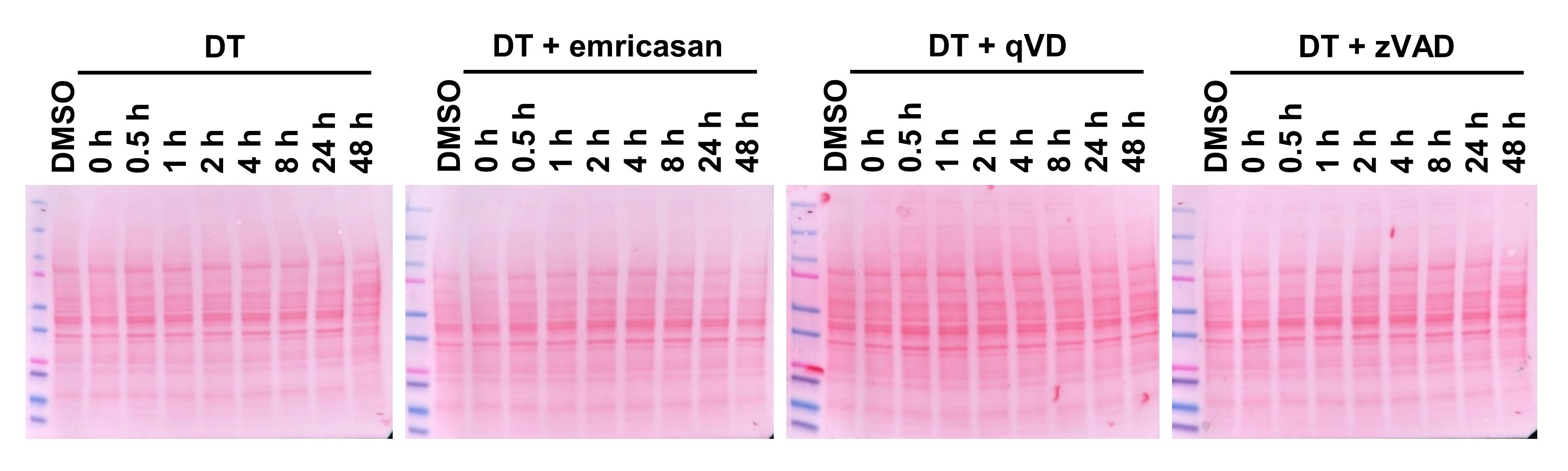

Supplement: Supplementary file 2 — Supplemental Video 3 [file ADVS-12-e07381-s007.jpg]

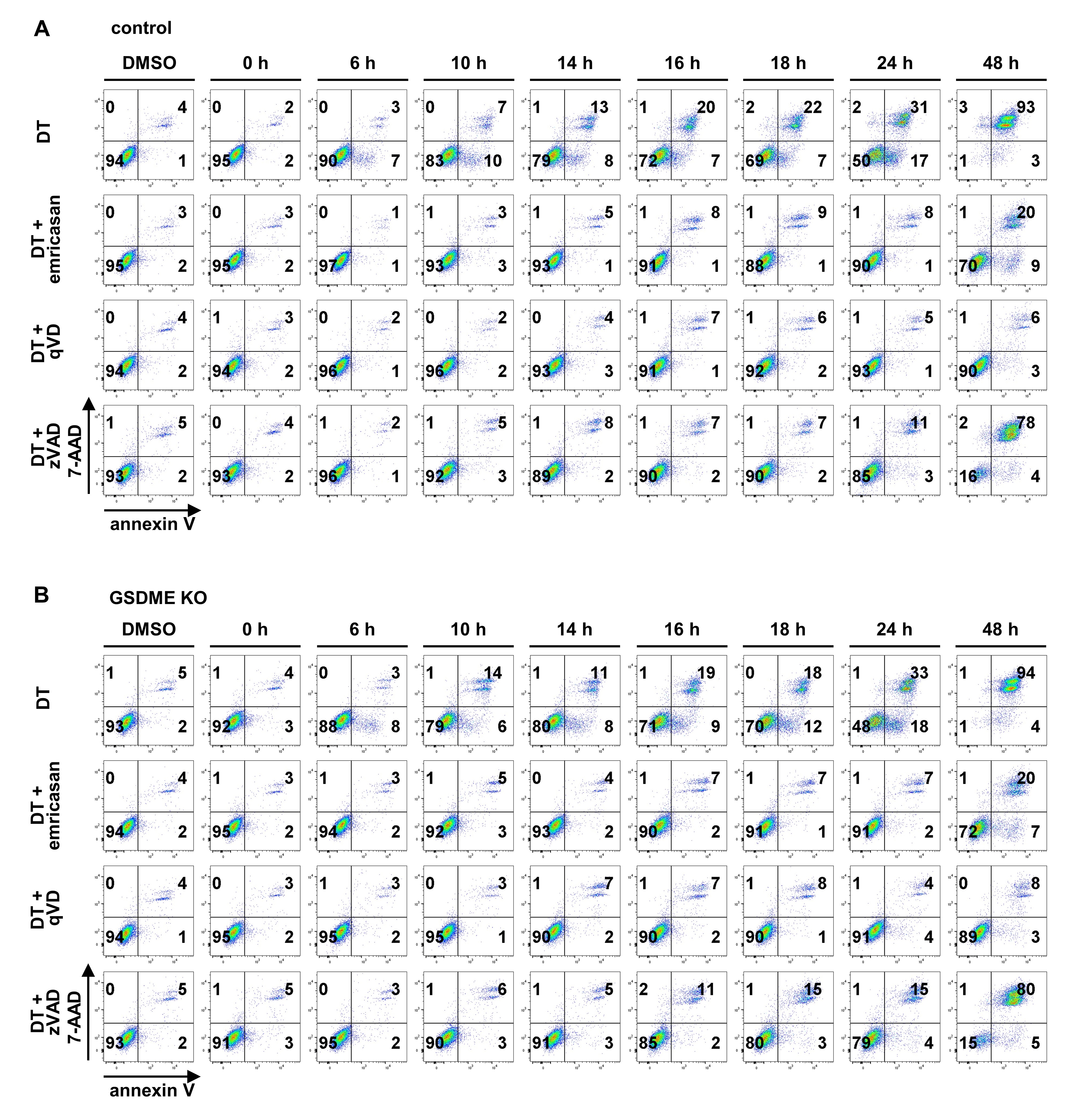

Supplement: Supplementary file 3 — Supplemental Video 3 [file ADVS-12-e07381-s006.jpg]

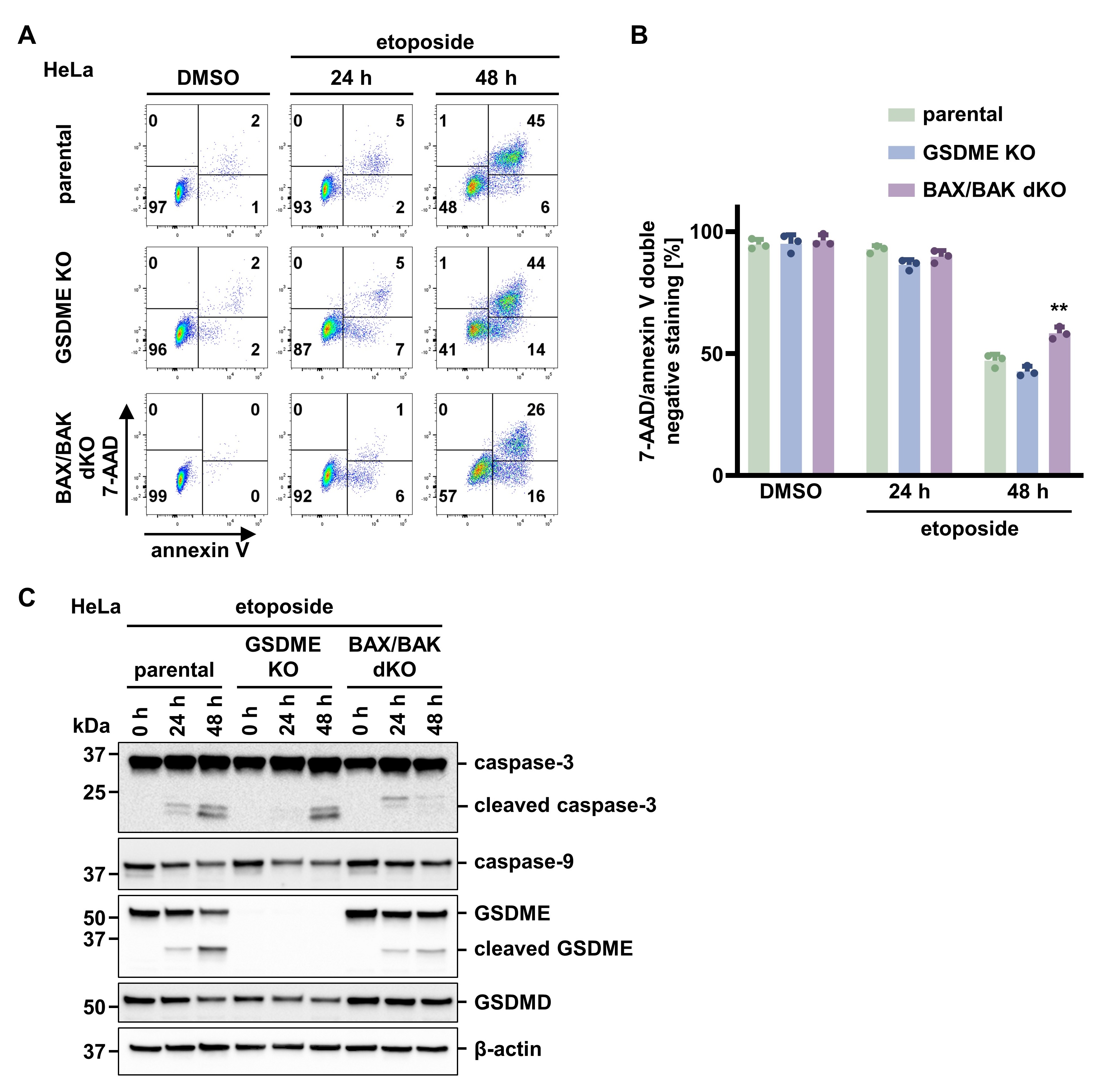

Supplement: Supplementary file 4 — Supplemental Video 3 [file ADVS-12-e07381-s003.jpg]
